# Antioxidant Potential of a Polyherbal Antimalarial as an Indicator of Its Therapeutic Value

**DOI:** 10.1155/2013/678458

**Published:** 2013-12-19

**Authors:** Protus Arrey Tarkang, Achille Parfait Nwachiban Atchan, Jules-Roger Kuiate, Faith Apoelot Okalebo, Anastasia Nkatha Guantai, Gabriel Agbor Agbor

**Affiliations:** ^1^Institute of Medical Research and Medicinal Plants Studies (IMPM), Yaoundé, Cameroon; ^2^Department of Pharmacology and Pharmacognosy, University of Nairobi, P.O. Box 19676-00202, Nairobi, Kenya; ^3^Department of Biochemistry, University of Dschang, P.O. Box 67, Dschang, Cameroon

## Abstract

*Nefang* is a polyherbal product composed of *Mangifera indica* (bark and leaf), *Psidium guajava, Carica papaya, Cymbopogon citratus, Citrus sinensis, and Ocimum gratissimum* (leaves), used for the treatment of malaria. Compounds with antioxidant activity are believed to modulate plasmodial infection. Antioxidant activity of the constituent aqueous plants extracts, *in vitro*, was evaluated using the 2,2-diphenyl-1-picrylhydrazyl (DPPH), total phenolic content (TPC), and ferric reducing antioxidant power (FRAP) methods and, *in vivo*, *Nefang* (100 and 500 mg kg^−1^) activity was evaluated in carbon tetrachloride-induced oxidative stressed Wistar rats. Superoxide dismutase, catalase activities, and lipid peroxidation by the malondialdehyde and total proteins assays were carried out. *P. guajava, M. indica* leaf, and bark extracts had the highest antioxidant properties in all three assays, with no statistically significant difference. Rats treated with the carbon tetrachloride had a statistically significant decrease in levels of triglycerides, superoxide dismutase, and catalase (*P* < 0.05) and increase in malondialdehyde activity, total protein levels, and liver and renal function markers, whereas rats treated with *Nefang* showed increased levels in the former and dose-dependent decrease towards normal levels in the later. These results reveal the constituent plants of *Nefang* that contribute to its *in vivo* antioxidant potential. This activity is a good indication of the therapeutic potential of *Nefang*.

## 1. Introduction

Molecular oxygen is an indispensable element for the life of aerobic organisms because it enables the formation of reactive oxygen species (ROS) which in small quantities are essential for many physiological processes. At high doses, the ROS are very toxic [1]. The main ROS in humans are superoxide anion (O_2_^•−^), hydrogen peroxide (H_2_O_2_^•^), radical hydroxide (OH^•−^) and the reactive nitrogen species (RNS), and nitric monoxide (NO). These are constantly generated within the cells upon exposure to xenobiotics, pollutants, ultraviolet rays, smoke, and some endogenous metabolites of the redox and respiratory chain during transfer of electrons [2].

Under normal physiological conditions, there is equilibrium between levels of intracellular ROS and the endogenous antioxidant system. The endogenous antioxidant system is composed of enzymes such as the superoxide dismutase (SOD), catalase (CAT), glutathione peroxidase (GPx), and glutathione (GSH). Over production of ROS beyond the antioxidant capacity of the physiological system gives way to oxidative stress. Excessive oxidative stress leads to the liberation of free radicals which leads to the oxidation of vital body molecules such as nucleic acids, lipids, glucosides, and proteins, by causing cellular lesions that could lead to necrosis and apoptosis [3, 4]. Oxidation of various biomolecules is also an etiological factor implicated in many age-related and chronic disease conditions such as cancer, diabetes, neurodegenerative diseases, and cardiovascular diseases [5–8], just to mention a few. Minimizing oxidative stress could therefore ameliorate many physical conditions and help prevent certain degenerative diseases caused by free radicals [9].

Antioxidants were rarely used in the past due to scarcity and high cost [10] but their use is presently on the rise with the preference being for those from natural sources as opposed to the synthetic ones (butyl-hydroxyl toluene, butyl-hydroxyl anisole, gallic acid), which have a relative innocuous ability [11]. Plants are a reservoir of novel antioxidant molecules especially those that have a high concentration of phenolic compounds [12]. Hence there is an increasing interest in natural antioxidants from food, particularly fruits and vegetables. Several epidemiologic reports suggest their protective effects against a growing list of diseases [13–15].

Oxidative stress is involved in the pathophysiology of malaria. Plasmodia digest hemoglobin which results in the production of heme. Heme triggers the production of ROS which are implicated in the pathophysiology of malaria [16, 17]and can lead to the development of anemia [18, 19]and apoptosis [20]. These are implicated in the pathogenic mechanisms triggered by the parasite [21] as well as free radical production [22] and antioxidant defenses [23] in host cells to abate the infection. Some antimalarial drugs such as artemisinin depend on the formation of free radical intermediates for the destruction of *Plasmodium* [24, 25].

Oxidative stress in malaria has been extensively reviewed [26] and conclusions were made about the fact that its role in the pathophysiology of malaria is a multifactorial phenomenon and represents an important aspect of the intricate and complex host-parasite relationship.

Plants and compounds with antioxidant activity may ameliorate the progress of malarial infection and probably prevent its sequelae. Experiments done in animals have shown that antioxidants prevented the development of cerebral complications [27].

*Nefang* is a polyherbal product composed of *Mangifera indica* (bark and leaf), *Psidium guajava, Carica papaya, Cymbopogon citratus, Citrus sinensis, *and *Ocimum gratissimum* (leaves) and it is used for the treatment of malaria in the South West Region of Cameroon. These constituent plants possess many biological activities and apart from *Citrus sinensis*, the others have been reported to have antiplasmodial activity [28]. It is probable that this polyherbal product and/or its constituent plant extracts contain metabolites with free radical scavenging activity and oxidant reducing capacity which may have beneficial effect in the management of malaria.

This study therefore aims at evaluating the *in vitro* and *in vivo* antioxidant potential of this polyherbal product as an indicator of its therapeutic value.

## 2. Materials and Methods

### 2.1. Ethical Considerations

Approval for the study was obtained from the Institutional Review Board of the Institute of Medical Research and Medicinal Plants Studies (IMPM), Yaoundé-Cameroon, and the Kenyatta National Hospital/University of Nairobi Ethics and Research committee, Nairobi, Kenya. The care and use of experimental animals described in the rationale and methodology of this research are in accordance with the goals, outcomes, and considerations defined in the Guide for Care and Use of Laboratory Animals by the Committee for the update of this guide, National Research Council of the National Academies [29]. The study was conducted in accordance with the protocol and GLP to ensure protection of all aspects of the ethical rights and welfare of research animals.

### 2.2. Collection and Extraction of Plant Material

Fresh bark and leaves of *Mangifera indica* and leaves of *Psidium guajava*, *Carica papaya*, *Cymbopogon citratus*, *Citrus sinensis*, and* Ocimum gratissimum* were harvested from their natural habitat in Cameroon in the months of July and August, 2011. Plant identification and voucher specimen referencing were done at IMPM herbarium in Yaoundé, Cameroon by a botanist. The freshly harvested plant parts were then air dried and pulverized.

For *in vitro* antioxidant evaluation, 10 mL of distilled water was added to 100 mg of each pulverized plant and heated in a water bath at 100°C for 90 min. This was allowed to cool down overnight and the aqueous supernatant was used immediately.

For *in vivo* studies, aqueous extraction of each plant material was carried out by percolation. Plant material was immersed in water for 4 h. The mac was transferred to a conical percolator for 72 h and the extract was filtered with a sieve of 80 *μ*m pore size [30]. The filtrate was first concentrated using a rotary evaporator and then dried up in an air oven at 60°C. The extract was weighed and stored in sealed plastic containers at 4–20°C for subsequent use.

### 2.3. Experimental Animal

Male Wistar rats (170–200 g) obtained from the animal house of IMPM were used for the *in vivo* antioxidant studies. They were housed in stainless steel wire mesh cages up to a maximum of 6 per cage, in a well-ventilated room with 12 h light/dark cycle, with free access to clean drinking water and food (standard rat feed). They were allowed to acclimatize for one week before experimentation. Plant extracts were administered orally.

### 2.4. Evaluation of *In Vitro* Antioxidant Activity

#### 2.4.1. 2,2-Di-phenyl-1-picryl-hydrazyl (DPPH) Radical Scavenging Activity Assay

DPPH radical scavenging activity was measured using the method described by Yen and Duh [31]. Twenty *μ*L of the aqueous plant extract was introduced to 2 mL methanol solution of DPPH (0.3 mM) and incubated at 37°C in the dark for 30 minutes. The extract was replaced by methanol for the control and catechin for the standard. Absorbance of the resulting solution was measured at 517 nm using a spectrophotometer. All the tests were performed in triplicate and the results averaged. The percentage DPPH radical scavenging activity was calculated by comparing the results of the test with those of the control (not treated with extract) using the following equation:

(1) Percentage radical scavenging activity=  (1−absorbance of testabsorbance of control)×100.  

#### 2.4.2. Estimation of Total Phenolic Compounds

Total soluble phenolic content in each plant extract was determined using the Folin-Ciocalteu reagent (FCR) according to the method described by Slinkard and Singleton [32]. Briefly, 0.1 mL of each aqueous plant extract was transferred to 100 mL Erlenmeyer flask then final volume was adjusted to 46 mL by addition of distilled water. After 3 min, 1 mL of FCR and 3 mL of Na_2_CO_3_ (2%) were added to this mixture. The mixture was then incubated for 2 hours at room temperature (25°C) then the absorbance was measured at 760 nm. All the tests were performed in triplicate and the results averaged. The concentration of total phenolic compounds in each extract was estimated as milligram of catechin equivalent by linear interpolation of a catechin standard curve.

#### 2.4.3. Ferric Reducing Antioxidant Power (FRAP) Assay

The ferric reducing antioxidant power of each extract was determined according to the method described by Benzie and Strain [33]. The FRAP reagent consisted of ten parts of acetate buffer (300 mM, pH 3.6), one part of 2,4,6-Tri (2-pyridyl)-s-triazine (TPTZ) (10 mM in 400 mM of HCl), and one part of ferric chloride (10 mM). Each extract (75 *μ*L) was transferred to a cuvette containing 2 mL of FRAP solution and after agitation absorbance was read after twelve minutes of incubation, at 593 nm. The ferric reducing antioxidant power in each extract was determined as milligram of catechin equivalent by linear interpolation of a catechin standard curve.

### 2.5. Evaluation of *In Vivo* Antioxidant Activity

#### 2.5.1. Animal Experiments

From previous *in vivo* antiplasmodial studies, we observed minimum and maximum activity at doses of 100 mg kg^−1^ and 500 mg kg^−1^ *BW*, respectively, giving the criteria dose determination in this study. Albino Wistarrats weighing 170–200 g were divided into four groups (A, B, C, and D) of 5 animals each. The first group was treated with the vehicle (corn oil). Oxidative stress was induced in the other three groups of animals by oral administration of 1 mL kg^−1^ *BW* carbon tetrachloride in corn oil (1 : 5 v/v). Two of these groups were treated with 100 and 500 mg kg^−1^ *BW* of *Nefang* aqueous extract by daily oral administration for 14 days. Twenty-four hours after the last dose, all the animals were anesthetized by intramuscular administration of Zoletil 50 (30 mg/Kg) + xylazine (5 mg/kg) for 5–10 minutes and blood was harvested by cardiac puncture into EDTA tubes after which they were sacrificed by cervical dislocation. The blood sample was centrifuged at 3000 rpm for 10 min. Supernatant plasma was collected for biochemical analysis. Physiological saline (0.9%) was then added to the packed cellular layer to double the volume. After mixing, it was centrifuged at 3000 rpm and the supernatant was discarded. This procedure was repeated thrice and the erythrocytes were then isolated and stored at −20°C for subsequent use. The liver, kidneys, and heart were harvested, cleaned of blood, and weighed.

#### 2.5.2. Determination of Biochemical Parameters

The plasma was subjected to biochemical analysis using standard analytical kits from Fortress Diagnostics Ltd, UK. The parameters that were determined included glucose (GLU), cholesterol (CHOL), triglycerides (TGY), alanine transaminase (ALT), aspartate transaminase (AST), blood urea nitrogen (BUN), and creatinine (CRE).

#### 2.5.3. Analysis for Oxidative Stress Markers

Red blood cells were used as a cellular model for evaluating the ability of antioxidants to cross the plasma membrane into living cells. Oxidative stress markers were analyzed in the RBC hemolysate. 

Superoxide Dismutase* (SOD)*. Activity was determined using the method described by Misra and Fridovich [34]. An aliquot of 0.2 mL of the haemolysate was added to 2.5 mL 0.05 M carbonate buffer (pH 10.2). This was allowed to equilibrate in a spectrophotometer. The reaction was started by adding 0.3 mL of freshly prepared 0.3 mM adrenaline to the buffered sample mixture. This was quickly mixed by inversion and placed in the spectrophotometer. The reference cuvette contained 2.5 mL of the buffer, 0.3 mL of the substrate, and 0.2 mL distilled water. Increase in absorbance at 480 nm was monitored every 30 seconds for 150 seconds against a blank.

SOD was calculated in units as the amount necessary to cause 50% inhibition of the oxidation of adrenaline to adrenochrome during one minute. 

Catalase* (CAT)*. Activity was determined using the method described by Sinha [35]. In six different tubes containing increasing concentration of hydrogen peroxide (0–640 *μ*M), 2 mL of acidified potassium dichromate was added. Each tube was shaken and heated at 100°C for 10 minutes then allowed to cool and optical density was read at 570 nm. A standard curve of absorbance against concentration of hydrogen peroxide was then plotted.

1 mL of diluted haemolysate (1 : 10 v/v) was then added to a tube containing 2 mL of hydrogen peroxide and 2.5 mL of 0.01 M phosphate buffer (pH 7.0) and mixed. From this mixture 1 mL of solution was withdrawn after every 30 seconds and transferred into 2 mL of acidified potassium dichromate for 120 seconds. The mixture was shaken and heated for 10 minutes at 100°C. After cooling the optical density was measured at 570 nm against a blank.

The activity was then calculated in units of catalase as the amount required to catalyze the reduction of 1 *μ*M of H_2_O_2_ in 1 minute. 

Lipid Peroxidation was measured by applying the malondialdehyde (MDA) assay method earlier described by Biswas et al. [36]. 0.4 mL of diluted haemolysate (1 : 10 v/v) was added to a tube containing 2 mL of acetic acid and 2 mL of a working reagent (NaOH + thiobarbituric acid). The tubes were incubated in an oven at 100°C for 20 minutes; the absorbance was then read at 532 nm against the blank. The concentration of lipid peroxides was then calculated from the molar extinction coefficient of 1.56 × 10^5^ mole^−1^ cm^−1^ as follows:

(2) Concentration of MDA=OD/lε   (ε=1.56×105 mole−1 cm−1),

where *ε* is the molar extinction coefficient and *l* is the length of the cuvette (cm). 

Total Proteins were estimated by the method described by Lowry et al. [37]. To different tubes containing 0.2 mL of diluted haemolysate and 0.2 mL bovine albumin (2 g/dL) standard, 2 mL of distilled water and 1 mL of working reagent 1 [1 vol (CuSO_4_ + NaK) + 100 vol (Na_2_CO_3_ + NaOH)] were added. The contents were mixed by inversion and incubated at room temperature for ten minutes. 0.25 *μ*L of 50% Folin Ciocalteu reagent was then added, mixed, and incubated at room temperature for ten minutes. Absorbance was then read at 570 nm against a blank.

The concentration of total proteins in the RBC haemolysate was determined from the equation below as follows:

(3) protein  (g/dL)=(Concentration  of  standardOD  of  standard)×OD  of  test  sample.

### 2.6. Statistical Analysis

Data was analyzed using SPSS 20.0 software. Variances were compared across groups using the one-way ANOVA and results were expressed as mean ± SD. The Waller-Duncan test was used to test for differences of means. Pearson's linear test was used to test for correlation. *P* values of less than 0.05 were considered to be statistically significant.

### 2.7. Results

#### 2.7.1. *In Vitro* Antioxidant Properties of the Plant Extracts

The results of the antioxidant activity of each extract as analyzed by the various methods are presented in Table 1. No significant difference was observed between the Total phenolic content, ferric reducing antioxidant power, and the radical scavenging activities of *P. guajava*, *M. indica* leaf and bark, and *O. gratissimum* aqueous extracts. The analysis of the radical scavenging activity by the DPPH assay revealed that *P. guajava* and *M. indica* (leaf and bark) had the greatest activity (>90% inhibition). Their total phenolic content ranged from 61.7 to 67.2 mg catechin equivalent/g extract. *O. gratissimum* had a moderate activity (59% inhibition) and total phenolic content of 34 mg catechin equivalent/g extract. *C. citratus, C. papaya*, and *C. sinensis* had very weak activity and low total phenolic content.

**Figure 1:**
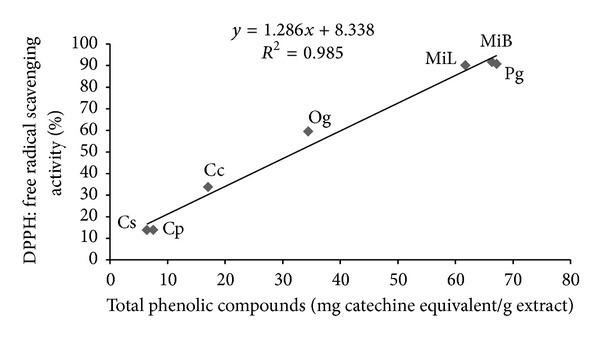
Pearson's correlation analysis between DPPH and FCR assays. Aqueous extracts: MiB—*Mangifera indica bark*, MiL—*Mangifera indica leaf*, Pg—*Psidium guajava*, Cp—*Carica papaya*, Cc—*Cymbopogon citratus*, Cs—*Citrus sinensis*, and Og—*Ocimum gratissimum* (leaves).

**Figure 2:**
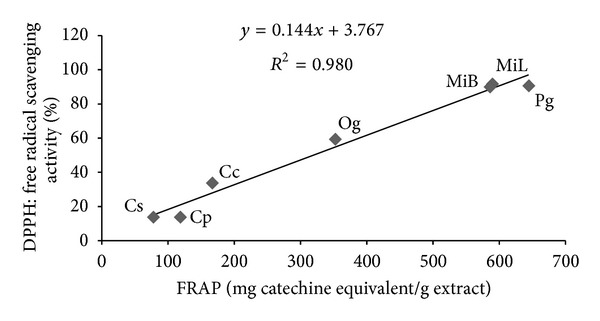
Pearson's correlation analysis between DPPH and FRAP assays. Aqueous extracts: MiB—*Mangifera indica bark*, MiL—*Mangifera indica leaf*, Pg—*Psidium guajava*, Cp—*Carica papaya*, Cc—*Cymbopogon citratus*, Cs—*Citrus sinensis*, and Og—*Ocimum gratissimum* (leaves).

**Figure 3:**
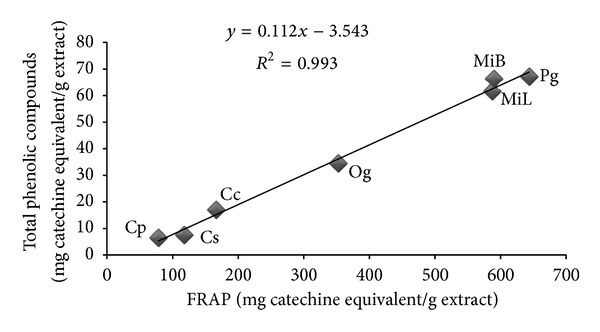
Pearson's correlation analysis between FCR and FRAP assays. Aqueous extracts: MiB—*Mangifera indica bark*, MiL—*Mangifera indica leaf*, Pg—*Psidium guajava*, Cp—*Carica papaya*, Cc—*Cymbopogon citratus*, Cs—*Citrus sinensis*, and Og—*Ocimum gratissimum* (leaves).

Figures 1, 2, and 3 summarize the relationship between DPPH, TPC, and FRAP antioxidant activity. A highly significant (*P* < 0.01) positive correlation was observed between the radical scavenging activity and total phenol content, the radical scavenging activity and antioxidant activity, and the total phenol content and antioxidant activity, with correlation indices of 0.985, 0.980, and 0.993, respectively. 

#### 2.7.2. *In Vivo* Antioxidant Activity: Effect of *Nefang* on CCl_4_-Intoxicated Rats****

**Figure 4:**
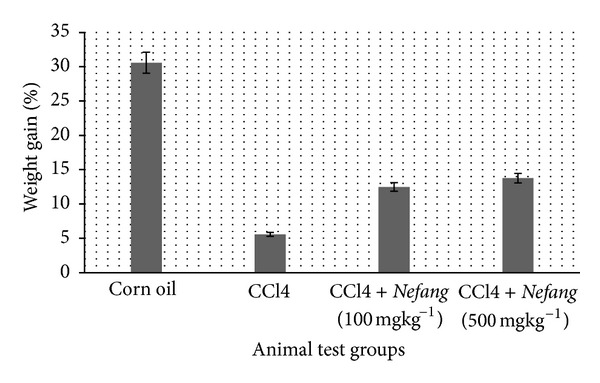
Percentage weight gain in CCl_4_-treated rats compared to nontreated rats. Bars represent means ± SD; *n* = 5.

*Effect on Physiological Parameters.* We observed a significant decrease in weight gain (*P* < 0.01) in all test groups (B, C, and D) compared to the untreated control group, with the group treated with carbon tetrachloride alone (B) showing the least gain in weight as summarized in Figure 4. This decrease in weight gain was probably due the significantly decreased water and food intake (*P* < 0.05). There was also hypertrophy of the liver in groups B and C rats when compared to the control. These effects are summarized in Table 2. During the experiment we also observed reduced mobility and change in colour of the fur in CCl_4_-treated rats which lasted along the experiment contrary to the test groups in which the fur regained its colour by the end of the experiment.

*Effect on Biochemical Parameters and Oxidative Stress Markers.* The effects of *Nefang* aqueous extract on biochemical parameters and oxidative stress markers are presented in Tables 3 and 4, respectively. These analyses revealed a significant decrease in CHOL (*P* < 0.05) and TGY level (*P* < 0.01) and a significant increase in BUN, CRE (*P* < 0.05), ALT, and AST (*P* < 0.01) in group B (CCl_4_-induced oxidative stressed) rats compared to the normal control group. In rats treated with *Nefang* (group C and D), we observed that at a dose of 100 mg kg^−1^, CHOL and ALT levels were abnormally high when compared to the untreated control group whereas at 500 mg kg^−1^, CHOL was restored to normal while ALT level decreased towards normal. However, *Nefang* prevented the increase in AST, BUN, and CRE while it increased TGY levels towards normal in a dose-dependent manner when compared to the untreated group. *Nefang* had no effect on the GLU level when compared to the untreated control group.

We also observed a significant decrease in SOD and CAT levels (*P* < 0.05), increase in TP and MDA (*P* < 0.05) in untreated control group (CCl_4_-induced oxidative stressed) compared to the normal control group. At a low *Nefang* dose of 100 mg kg^−1^, levels of SOD, CAT, and MDA remained comparable to that of the untreated control groups. However, at a higher dose of 500 mg kg^−1^, we observed a significant increase in SOD (*P* < 0.05) and a decrease in MDA (*P* < 0.05) levels compared to the untreated control group, showing that this activity was dose-dependent.

## 3. Discussion

Antioxidants are chemically diverse, therefore their separation and quantification with regard to their source are very demanding. Due to the increasing interest in antioxidants, there has been an increase in *in vitro* biological assays for the estimation of total antioxidant activity from different sources. In this study, the aim was to use *in vitro* biological methods to compare the antioxidant activities of the constituent plant extracts of *Nefang *while the *in vivo* antioxidant activity evaluates the potential protective role of *Nefang* against oxidative stress induced by CCl_4_. The antioxidant capacity of whole blood is contained predominantly in circulating RBC whose effective and powerful antioxidant system has been highlighted as a potential major advantage for using RBCs to study ROS generation [38].

Among the constituent plants of *Nefang*, the leaf extracts of *P. guajava, M. indica* (leaf and bark), and *O. gratissimum* exhibited potent *in vitro* antioxidant activity in DPPH radical scavenging and FRAP assays, which correlated with their high total phenolic content. The correlation coefficients between the assays were very strong and were close to 1.

2,2-Diphenyl-1-Picryl-Hydrazyl (DPPH) is a commonly employed assay in antioxidant studies of plant extracts or specific compounds over a short time period, since it provides information on the reactivity of extracts with a stable free radical. The efficacies of antioxidants are usually associated with their ability to inhibit oxidative damage by scavenging free radicals [39]. Total phenolic content estimation is a simple reproducible assay to estimate the total phenolic antioxidants in an extract [15]. Phenolic compounds are considered to be the most important antioxidative plant components [40] and the antioxidant activity of plant extracts correlates with the content of their phenolic compounds [41]. FRAP is a simple inexpensive method to measure the total antioxidant reducing power of plant extracts in the RBC [42]. The fact that all the constituent plants of *Nefang* possess radical scavenging activities, TPC, and FRAP (with exception of *Citrus sinensis*, *Cymbopogon citratus*, and *Carica papaya* leaves with comparatively weak activity) suggests that *Nefang* may possess the antioxidant potential to combat oxidative stress induced during malaria infection or treatment.

In the present study, carbon tetrachloride was used to induce oxidative stress so as to evaluate the potential of *Nefang* as *in vivo* antioxidant. Carbon tetrachloride is widely used as a toxicant for experimental induction of liver toxicity in laboratory animals, because it induces lipid peroxidation in experimental animals within few minutes of administration. Accumulation of CCl_4_ in the parenchyma cells results into hemolytic cleavage producing trichloromethyl (CCl_3_*) and the chloride (Cl*) free radicals due to the activating effect of cytochrome P_450_ enzymes. The CCl_3_* alkylates cellular proteins and other molecules with a simultaneous attack on polyunsaturated fatty acids in the presence of oxygen, forming lipid peroxides and leading to severe damage to some visceral organs [43–46] and the massive generation of free radicals.. Thus administration of CCl_4_ at specific doses produces marked organ damage, as evidenced by a significant (*P* < 0.05) increase in renal (ALT, AST) and hepatic (BUN, CRE) enzymes and lipid peroxidation. It causes hepatotoxicity, characterized by an increase in organ mass. The relative liver weight of all the groups treated with CCl_4_ increased as compared to the normal control group animals. However, this increase in liver weight was reduced in *Nefang*-treated groups and the effect was dose dependent, showing protective nature of *Nefang*. The rats treated with 500 mg kg^−1^ *BW* of *Nefang* recorded the least gain in body weight. This corroborate with the reduced appetite characterized by decreased food and water intake.

*Nefang* seemed to offer dose-dependent protective effect because the levels of the renal and hepatic enzymes were lower in extract-treated rats as compared to the untreated control group. This was achieved by inhibiting the effect of CCl_4_ in the plasma.

Oral administration of CCl_4_ decreased CHOL and TGY levels as reported earlier by Stern et al. [47]. Cholesterol has been reported to modulate the lipid bilayer membrane fluidity and other physiological processes [48]. CCl_4_ causes peroxidative degradation of the cellular membrane leading to functional morphological changes and loss of functional integrity of the membranes [15]. By restoring the cholesterol level to normal, it is evident that *Nefang* may modulate the role of cholesterol in this and other physiological processes.

The human body has an effective defense system against free radical induced damage, which consists of endogenous antioxidant enzymes (e.g., CAT and SOD) and nonenzymatic enzymes (e.g., GSH). SOD and CAT form part of the enzymatic oxidative system of RBCs while MDA is an end product of lipid peroxidation in the liver and other tissues. The level of lipid peroxide is a measure of membrane damage alterations in its structure function and is measured by the level of MDA. Erythrocytes are more vulnerable to lipid peroxidation [49]. SOD is a metalloprotein enzyme involved in antioxidant defense, which acts by lowering the steady state level of O_2_^•−^ while catalase protects cells against radical toxicity by catalyzing the decomposition of hydrogen peroxide to water and molecular oxygen. Decreased levels of SOD and CAT in CCl_4_-treated rats may be due to the overuse of these enzymatic antioxidants or to the cross linking of these enzymes with MDA, causing an increase in the level of superoxide anion which in turn increases lipid peroxidation [50]. In the present study, levels of SOD and CAT decreased and MDA levels increased in untreated control group when compared to the normal control group. This provided evidence of oxidative stress leading to enhanced lipid peroxidation, tissue damage, and collapse of the antioxidant defense mechanism against free radicals. However, upon administration of *Nefang *at a high dose, these parameters did not show any stress to the antioxidative defense system, though at a low dose, levels of these parameters were comparable to that in untreated rats. This could be explained by the potent antioxidant activity or increased enzyme expression. *Nefang* therefore provided antioxidant effects.

The TP estimation gives the protein content and reflects the antioxidant capacity. The increased TP level in CCl_4_-induced oxidative stress rats (group B) may be due to dehydration [51]. TP was dose dependently restored to normal in rats treated with the extract, revealing that *Nefang* may help boost the potential of the antioxidant system through its high phenolic content as evidenced in the *in vitro* studies.

Given that there is laboratory evidence that antioxidants ameliorate symptoms of malaria [27], it is highly likely that the potent antioxidants found in *Nefang* contribute to effectiveness in the management of malaria when used traditionally. This may explain why multiple herbs are included in the formulation because each might serve a different purpose.

## 4. Conclusion

This study has generated substantial evidence of the *in vitro* antioxidant effect of the constituent plants and *in vivo* antioxidant effects of *Nefang*. The aqueous leaf extracts of *P. guajava* and *M. indica *(leaf and bark) had the highest *in vitro* antioxidant capacity. There is a very strong positive correlation between the results obtained by DPPH, TPC, and FRAP. *Nefang* may contribute to improve the antioxidant status of oxidative stress-associated complications including malaria. The study of the antimalarial properties and antioxidant effect of *Nefang* in *P. falciparum *infected mice is being carried out.

## Figures and Tables

**Table 1:** *In vitro* antioxidant activity of constituent plant extracts of *Nefang*.

Aqueous extract (10 mg/mL)	DPPH inhibition of radical activity (%)	Total phenolic content (TPC) (mg catechine equivalent/g extract)	FRAP (mg catechine equivalent/g extract)
*Mangifera indica bark *	90.11 ± 0.73^(3)^	66.33 ± 1.92^(3)^	587.08 ± 8.01^(3)^
*Mangifera indica leaf *	90.76 ± 0.10^(2)^	61.73 ± 2.68^(2)^	590.17 ± 6.76^(2)^
*Psidium guajava leaf *	91.65 ± 0.38^(1)^	67.15 ± 10.57^(1)^	644.17 ± 25.69^(1)^
*Carica papaya leaf *	13.88 ± 3.10^(6)^	6.41 ± 0.90^(7)^	117.95 ± 31.23^(6)^
*Cymbopogon citratus leaf *	33.74 ± 4.01^(5)^	17.03 ± 0.39^(5)^	166.72 ± 78.37^(5)^
*Citrus sinensis leaf *	13.81 ± 4.42^(7)^	7.59 ± 0.98^(6)^	78.09 ± 0.76^(7)^
*Ocimum gratissimum leaf *	59.52 ± 7.93^(4)^	34.42 ± 1.74^(4)^	353.00 ± 9.43^(4)^

Results presented as mean ± SD; *n* = 3. In the same column, values that designated different superscripts are significantly different. ^( )^Ranking.

**Table 2:** Effect of *Nefang* on selected physiologic parameters.

Physiologic parameters	Group A (corn oil)	Group B ( CCl_4_)	Group C (CCl_4_ + 100 mg/kg *Nefang*)	Group D (CCl_4_ + 500 mg/kg *Nefang*)
Food intake (g)	125.92 ± 16.64	85.69 ± 30.04*	93.53 ± 33.39*	94.30 ± 29.11*
H_2_O intake (mL)	88.76 ± 20.1	47.69 ± 27.43*	49.61 ± 24.95*	51.46 ± 26.81*
Relative weight of Heart (g)	0.33 ± 0.19	0.33 ± 0.19	0.29 ± 0.02	0.32 ± 0.03
Relative weight of liver (g)	3.36 ± 0.39	4.78 ± 0.49*	4.37 ± 0.25*	4.02 ± 0.3
Relative weight of kidneys (g)	0.34 ± 0.01	0.36 ± 0.01	0.36 ± 0.01	0.39 ± 0.01

Results presented as mean ± SD; *n* = 5; significant difference: *P < 0.05, **P < 0.01.

**Table 3:** Effect of *Nefang* on biochemical parameters in CCl_4_-treated Wistar rats.

Biochemical parameters	Group A (corn oil)	Group B ( CCl_4_)	Group C (CCl_4_ + 100 mg/kg *Nefang*)	Group D (CCl_4_ + 500 mg/kg *Nefang*)
GLU (mg/dL)	124.07 ± 5.69	151.87 ± 5.03*	160.04 ± 4.31	154.30 ± 10.69
CHOL (mg/dL)	86.98 ± 7.60	74.92 ± 7.69*	135.71 ± 23.80^##^	91.42 ± 5.03
TGY (mg/dL)	227.47 ± 5.89	142.28 ± 25.27**	154.89 ± 18.60^#^	166.01 ± 3.35^#^
ALT (UI/L)	16.97 ± 2.05	97.38 ± 5.26**	126.67 ± 12.08^##^	54.78 ± 2.85^##^
AST (UI/L)	35.52 ± 3.94	69.52 ± 1.29**	63.77 ± 7.01^#^	54.84 ± 7.75^#^
BUN (mg/dL)	35.63 ± 3.01	40.73 ± 1.50*	39.08 ± 3.32	33.12 ± 0.38^#^
CRE (mg/dL)	0.77 ± 0.09	0.88 ± 0.83*	0.88 ± 0.04	0.76 ± 0.05^#^

GLU: glucose, CHOL: cholesterol, TGY: triglycerides, ALT: alanine aminotransferase, AST: aspartate aminotransferase, BUN: blood urea nitrogen, CRE: creatinine. results presented as mean ± SD; *n* = 5; significant difference: compared to normal control—*P < 0.05, **P < 0.01, compared to untreated control— ^#^ *P* < 0.05, ^##^ *P* < 0.01.

**Table 4:** Effect of *Nefang* on oxidative stress markers in CCl_4_-treated wistar rats.

Biochemical parameters	Group A (corn oil)	Group B ( CCl_4_)	Group C (CCl_4_ + 100 mg/kg *Nefang*)	Group D (CCl_4_ + 500 mg/kg *Nefang*)
SOD (UI/mg)	8.15 ± 0.35	6.77 ± 0.19*	8.54 ± 0.53	9.56 ± 0.43^#^
CAT (UI/mg)	0.1 ± 0.20	0.04 ± 0.00**	0.14 ± 0.04^#^	0.12 ± 0.30^#^
MDA (*µ*Mol/L)	1.15 ± 0.30	1.18 ± 0.19*	1.17 ± 0.07	1.07 ± 0.05^#^
TP (mg/mL)	24.05 ± 1.06	27.72 ± 1.06*	20.51 ± 1.53^#^	24.15 ± 2.8

SOD: superoxide dismutase, CAT: catalase, MDA: malondialdehyde, TP: total proteins.Results presented as mean ± SD; *n* = 5; significant difference: compared to normal control—*P < 0.05, **P < 0.01, compared to untreated control—^#^ *P* < 0.05, ^##^ *P* < 0.01.
